# Paediatric morbidity and mortality in Sierra Leone. Have things changed after the 2014/2015 Ebola outbreak?

**DOI:** 10.12688/f1000research.18552.2

**Published:** 2020-01-09

**Authors:** Tom Sesay, Olga Denisiuk, Rony Zachariah

**Affiliations:** 1 Child Health and Immunization Programme, Ministry of Health and Sanitation, Freetown, Sierra Leone; 2 Alliance for Public Health, Kiev, Ukraine; 3 Special Programme for Research and Training in Tropical Diseases (WHO/TDR), Geneva, Switzerland

**Keywords:** Sustainable Development Goals, Outbreak response, Universal Health Coverage, Operational Research, SORT IT

## Abstract

**Background:** Sierra Leone was severely affected by the 2014/2015 Ebola outbreak which is likely to have had longer term repercussions on the health system including on paediatric morbidity and mortality. We thus assessed under-five morbidity and mortality for malaria, acute respiratory Infections (ARI)/pneumonia, watery diarrhoea and measles during the post-Ebola period in Sierra Leone and compared this with the pre- and intra-Ebola periods.

**Methods: **This was a retrospective cross-sectional study using program data from the District Health Information system (DHIS2) and sourced from 14 districts in Sierra Leone. It included under-five children from 1,250 health facilities country-wide. Study periods included: before (June 1 ^st^, 2013-April 30 ^th^, 2014); during (June 1 ^st^, 2014-April 30 ^th^, 2015); and after Ebola (June 1 ^st^, 2016-April 30 ^th^, 2017).

**Results:** Malaria, ARI/pneumonia and diarrhoea consultations declined during Ebola but recovered to pre-Ebola levels in the post-Ebola period.  During the post-Ebola period, there was a highly significant reduction in case-fatality for the first three morbidities compared to the pre-Ebola period ( *P*<0.0001). Average number of measles cases increased from 48/month in the pre-Ebola period to 568/month (12-fold increase) post-Ebola. Although there was no difference in measles case-fatality between the pre- and post-Ebola periods, case-fatality post-Ebola was significantly lower than during Ebola (Relative Risk: 0.05, 95% confidence interval 0.02-0.15, *P*<0.0001).

**Conclusions:** Consultations for under-five children at health facilities in Sierra Leone recovered to pre-Ebola levels and case-fatality for common childhood illnesses declined significantly. This is a change for the better. However, the high level of reported measles cases in the post-Ebola period indicates gaps in immune status and needs focused attention.

## Introduction

In 2017, a cross-sectional study ^1^ documented country-wide morbidity for four common childhood illnesses: malaria, acute respiratory infections (ARI)/pneumonia, watery diarrhoea and measles. There were two main findings. First, during the Ebola outbreak, health facility visits for malaria, ARI/pneumonia and watery diarrhoea dropped significantly nation-wide, without returning to pre-Ebola levels post-outbreak. As these morbidities have similar symptom patterns as Ebola, people may have avoided accessing formal health services to avoid being considered “an Ebola case”. Second, measles cases increased dramatically by six-fold during Ebola and the immediate post-Ebola periods. This was attributed to cessation of measles vaccination activities during the Ebola outbreak.

The outbreak was declared over in November 2015. Since then, there have been considerable investments into the health system by Government and development partners. One of the limitations of the 2017 study ^1^ was that it only included the immediate six-month period after the Ebola outbreak, which might have been too early to assess health system recovery or possible improvement. It is now expected that the country would have fully recovered from the outbreak, but there has been no formal evaluation in this regard. 

We thus conducted a similar country-wide study assessing morbidity and mortality for the same childhood illnesses using a longer post-Ebola period and compared this data with the pre- and intra Ebola periods.

## Methods

This was a retrospective analysis using routine program data from the District Health Information system (DHIS2) and sourced from all 14 districts in Sierra Leone (see Underlying data ^2^).

The study setting was described in detail before ^1^. In brief, the health infrastructure is tiered into tertiary hospitals, district hospitals and Peripheral Health Units (PHUs). PHUs include Community Health Centres (CHCs), Community Health Posts (CHPs) and Maternal and Child Health Posts (MCHPs). The Ministry of Health and Sanitation provides free primary health care for children under five across 1,250 health facilities nationwide.

The study population included all children under-five years attending public health facilities nationwide. No children were excluded.

Study periods included: before (June 1 ^st^ 2013-April 30 ^th^ 2014); during (June 1 ^st^ 2014-April 30 ^th^ 2015); and after Ebola (June 1st 2016-April 30th 2017).

We exported data on health facility visits and mortality for malaria, ARI/pneumonia, watery diarrhoea and measles from the DHIS2 to Microsoft excel (2016) for analysis. Differences between groups were assessed using Pearson’s X ^2^ test (Chi square) for the case fatality and t-tests for the average monthly consultations. Levels of significance were set at *P* ≤ 0.05.

### Ethics and consent

Ethics approval was obtained from the Sierra Leone Ethics and Scientific Review Board (dated 14 December 2018) and the Union Ethics Advisory Group (International Union against Tuberculosis and Lung Disease, Paris, France; EAG number: 68/18). As the study used anonymous data, there was no need for informed consent.

## Results

### Country-wide trend in out-patient consultations for under-five morbidities

**Figure 1.**
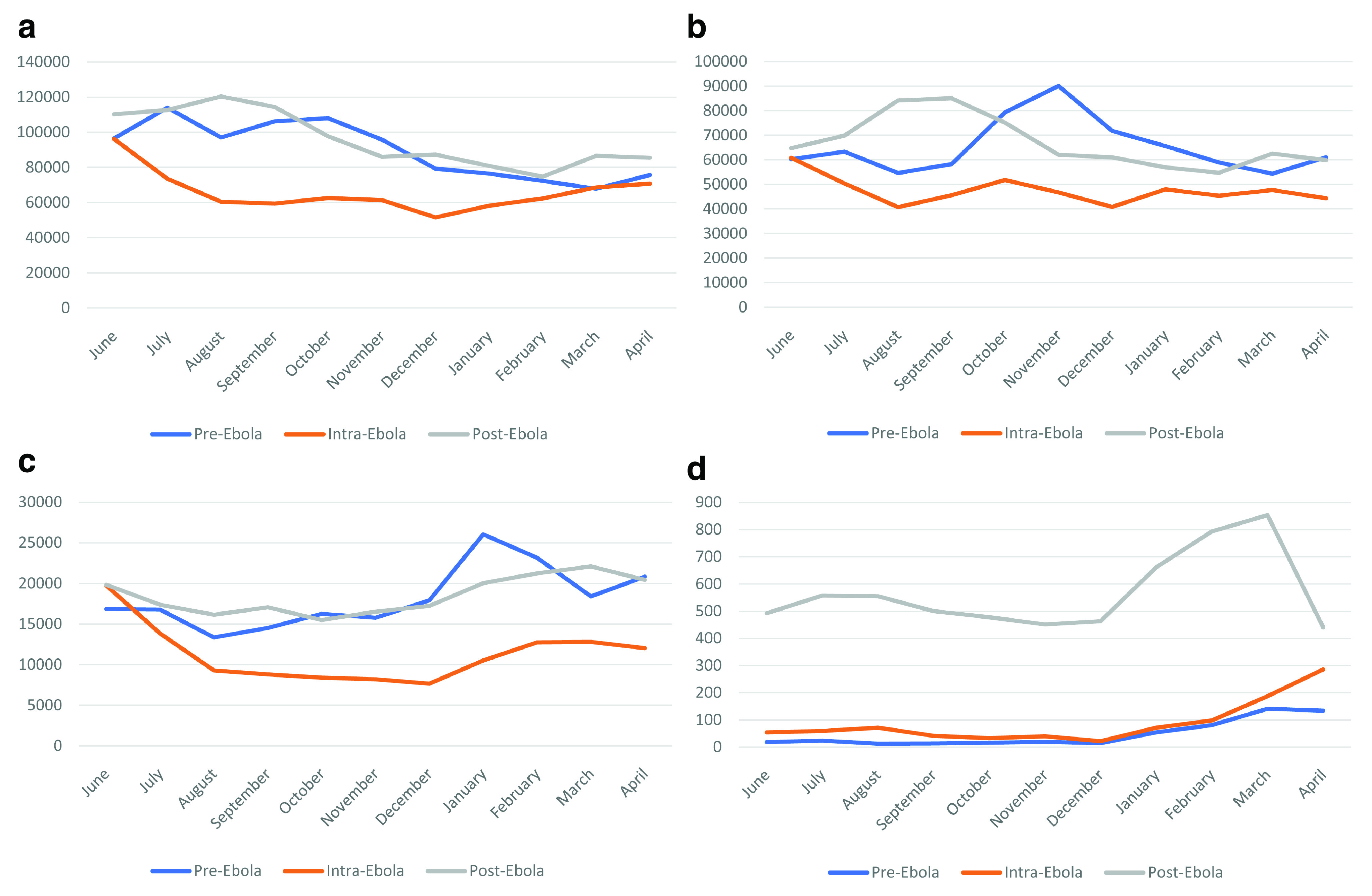
Trends in consultations for childhood morbidities in the pre-Ebola (1 June 2013–30 April 2014), intra-Ebola (1 June 2013–30 April 2014), and post- Ebola (1 June 2016–30 April 2017) periods, Sierra Leone. **a**): Malaria consultations in the pre-Ebola, intra-Ebola and post-Ebola periods. **b**) ARI/Pneumonia consultations in the pre-Ebola, intra-Ebola and post-Ebola periods. **c**) Watery diarrhea consultations in the pre-Ebola, intra-Ebola and post-Ebola periods. **d**) Measles consultations in the pre-Ebola, intra-Ebola and post-Ebola periods.

Figure 1 shows country-wide trends in outpatient consultations for malaria, ARI/pneumonia, watery diarrhoea and measles. Consultations followed a seasonal pattern with an overall decline during Ebola. In the post-Ebola period (assessed six months after the end of the outbreak), consultations reached pre-Ebola levels. In contrast, measles increased during the last six months of the Ebola outbreak and this trend continued into the post-Ebola period. Average numbers of measles cases were 48/month in the pre-Ebola period, increasing to 87/month in the Ebola period and 568/month (12-fold increase) post-Ebola. Measles cases peaked in March 2017 with 853 cases.

Table 1 shows that the observed differences of the pre-Ebola period relative to both the intra-Ebola and post-Ebola periods were statistically significant for all morbidities.

**Table 1.** Difference in mean monthly consultations for four morbidities between the pre-Ebola (1 June 2013–30 April 2014) period and the intra-Ebola (1 June 2013–30 April 2014) and post- Ebola (1 June 2016–30 April 2017) periods, Sierra Leone.

Condition	Pre/Intra-Ebola			Pre/Post-Ebola		
	Difference	95% CI	*P*-value	Difference	95% CI	*P*-value
**ARI/Pneumonia**	-17,771	(-17,804 to -17,738)	p<0.0001	1,661	(1,628 to 1,693)	p<0.0001
**Malaria**	-24,071	(-24,060 to -23,973)	p<0.0001	6,117	(6,093 to 6,161)	p<0.0001
**Measles**	39	(31 to 47)	p<0.0001	520	(508 to 532)	p<0.0001
**Watery Diarrhoea**	-6,900	(-6,926 to -6,874)	p<0.0001	320	(301 to 339)	p<0.0001

CI: Confidence Interval

### Morbidity and case-fatality for four under-five morbidities

Table 2 shows numbers of cases, deaths and case-fatality (per 1,000) for malaria, ARI/pneumonia, watery diarrhoea and measles. During the post-Ebola period, there was a highly significant reduction in case-fatality for the first three morbidities compared to the pre-Ebola period ( *P*<0.0001).

**Table 2.** Morbidity and case-fatality for four under five morbidities before, during and after the Ebola outbreak in Sierra Leone ^1^.

	Pre-Ebola	Ebola	Post-Ebola	*P*-value ^2^
	n	n	n	
**Malaria**				
Cases	989,068	724,881	1056354	
Deaths	2,564	1,205	2,112	
Deaths/1000	2.6	1.7	2.0	<0.0001
**ARI/Pneumonia**				
Cases	717,345	521,860	735,836	
Deaths	849	794	568	
Deaths/1000	1.2	1.5	0.8	<0.0001
**Watery Diarrhoea**				
Cases	200,006	124,100	203,520	
Deaths	361	150	242	
Deaths/1000	1.8	1.2	1.2	<0.0001
**Measles**				
Cases	525	962	6,245	
*Deaths*	1	16	6	
Deaths/1000	1.9	16.6	1.0	0.5

^1^ Pre-Ebola: June 1 ^st^ 2013 – April 30 ^th^ 2014; Ebola: June 1 ^st^ 2014 – April 30 ^th^ 2015; Post-Ebola: June 1 ^st^ 2016 – April 30 ^th^ 2017. ^2^ Chi-square comparing the pre-Ebola and post-Ebola periods.

For measles, there was a total of 525 cases pre-Ebola, 962 cases during Ebola and 6,245 cases post-Ebola. Although there was no difference in measles case-fatality between the pre- and post-Ebola periods, case-fatality post-Ebola was significantly lower than during Ebola (Relative Risk: 0.05, 95% confidence interval 0.02–0.15, *P*<0.0001).

## Discussion

This study shows that health facility consultations for malaria, ARI/Pneumonia and watery diarrhoea recovered to pre-Ebola levels and were accompanied by significant country-wide reductions in case-fatality compared to the pre-Ebola period. Despite a dramatic increase in measles cases post-Ebola, there was a significant mortality reduction, suggesting overall improvements in clinical care.

A study strength is that we included data from 1,250 health facilities and for similar periods before, during and after the outbreak. A limitation is that our data did not include private health facilities resulting in possible underestimation of disease burden. In addition, our post-Ebola period was limited to 2017, which may not be representative of long-term changes in systems strengthening.

There were two key findings. First, the reductions in case fatality from malaria, ARI/pneumonia and watery diarrhoea could be associated with post- Ebola health system investments with improved health seeking behaviour. The post-Ebola recovery plan ^3^ of the Government of Sierra Leone with enhanced financial, technical and training support from partners may also have contributed. Furthermore, community health worker activities including early identification and referrals of ill children were promoted which in turn would contribute to reducing mortality.

Second, increased measles cases during and after Ebola could be attributed to vaccination service cessation during Ebola in line with the recommendation to avoid invasive procedures as a way of minimizing Ebola-related occupational risks ^4^. Many children would have missed their measles vaccination, resulting in a reduction in herd immunity as well as an accumulation of unvaccinated children. Measles coverage among children under two years in 2017 (post-Ebola) stood at 80% ^5^ while pre-Ebola this was at a low 78.6% ^6^. This implies that measles vaccination coverage was already below the desired level prior to Ebola, worsened during Ebola and remained below desired levels after Ebola. The decline in immunization services during the Ebola period may have triggered a measles outbreak; however the resumption of immunization services would have contributed to the reduction of the cases in the post-Ebola period. This calls for strategies to increase immunisation coverage to at least 95% ^7, 8^ and to increase in the coverage of the second measles dose taken at 15 months of age.

Future research may consider including additional time periods of 2018-2019 to understand the longer term changes of health systems strengthening efforts on service delivery.

In conclusion, consultations of under-five children at health facilities in Sierra Leone recovered to pre-Ebola levels and case-fatality for common childhood illnesses declined significantly. This is a change for the better. However, the high level of reported measles cases in the post-Ebola period needs focused attention.

## Data availability

### Source data

The Sierra Leone Health Management Information Systems, the District Health Information System 2 (DHIS2), is accessible with a Ministry of Health and Sanitation (MOHS) login through https://sl.dhis2.org/. The Directorate of Policy, Planning, and Information (DPPI) can be contacted to arrange access through Dr. Francis Smart ( drfsmart@gmail.com), Director, DPPI, MOHS.

### Underlying data

Repository: Dataset 1. Sesay_Tom_SORTIT2_paed_data. https://doi.org/10.17605/OSF.IO/SYP7G ^2^

This project contains the following underlying data:

- Sesay_T_casefatality_data.xlsx (case fatality data)
- Sesay_T_morbidity_data.xlsx (morbidity data)
- Sesay_T_paed_datadictionary.xlsx (data dictionary)

Data are available under the terms of the Creative Commons Zero "No rights reserved" data waiver (CC0 1.0 Public domain dedication).
