# Supplementary material for: Paediatric morbidity and mortality in Sierra Leone. Have things changed after the 2014/2015 Ebola outbreak?
Source: F1000Res. 2020 Jan 9;8:796. Originally published 2019 Jun 6. [Version 2] doi: 10.12688/f1000research.18552.2 (PMC7676393; doi:10.12688/f1000research.18552.2)
Supplement: Peer review history

# Peer review: Reviewer response for version 2

**DOI:** 10.5256/f1000research.23885.r74704

**Published:** 2020-12-15

**Authors:** Vic Arendt

**Affiliations:** 1 Service National des Maladies Infectieuses, Centre Hospitalier de Luxembourg, Luxembourg City, Luxembourg

This article reports the impact of the West African Ebola outbreak in Sierra Leone (from early 2014-late 2015) on the number of consultations for common childhood diseases at 1250 health facilities as well as case fatality rates for these diseases.

 The conclusions are highly relevant regarding the recovery of the health system and the attendance to these facilities after the end of the Ebola epidemic.

 Regarding the figures of mortality, the conclusion that the case fatality between pre and post-ebola periods decreased significantly is clear.

 Strange enough, however, the mortality from malaria and diarrheal diseases from the pre to intra-ebola period also decreased a lot and it would be useful to comment on this. Could this be because of mass drug administration for malaria during the outbreak ^1^^ ^or could it be due to underreporting fever-related death due to fear of being suspected as Ebola cases or other explanations? Or decreased social contacts during the epidemic leading to a decrease in diarrheal diseases?

Is the work clearly and accurately presented and does it cite the current literature?

Yes

If applicable, is the statistical analysis and its interpretation appropriate?

Yes

Are all the source data underlying the results available to ensure full reproducibility?

Yes

Is the study design appropriate and is the work technically sound?

Yes

Are the conclusions drawn adequately supported by the results?

Yes

Are sufficient details of methods and analysis provided to allow replication by others?

Yes

Reviewer Expertise:

infectious diseases

I confirm that I have read this submission and believe that I have an appropriate level of expertise to confirm that it is of an acceptable scientific standard.

## References

[1] : Impact of the Mass Drug Administration for malaria in response to the Ebola outbreak in Sierra Leone. *Malar J*.2016;15: 10.1186/s12936-016-1493-1 480 10.1186/s12936-016-1493-1 27646649PMC5028945

# Peer review: Reviewer response for version 2

**DOI:** 10.5256/f1000research.23885.r74709

**Published:** 2020-11-30

**Authors:** Lutgarde Lynen

**Affiliations:** 1 Department of Clinical Sciences, Institute of Tropical Medicine Antwerp, Antwerp, Belgium

This is a follow-up study on the impact of the West-African Ebola outbreak on health seeking behavior for common childhood illnesses and their case-fatality in Sierra Leone.

 It is important to report on these indirect impacts of outbreaks on population health and hopefully lessons can be learnt for future epidemics, in how to mitigate these.

 The authors have sufficiently addressed the comments of the reviewers in the first round.

 The results are clear and are correctly discussed. I have no further comments.

Is the work clearly and accurately presented and does it cite the current literature?

Yes

If applicable, is the statistical analysis and its interpretation appropriate?

Yes

Are all the source data underlying the results available to ensure full reproducibility?

Yes

Is the study design appropriate and is the work technically sound?

Yes

Are the conclusions drawn adequately supported by the results?

Yes

Are sufficient details of methods and analysis provided to allow replication by others?

Yes

Reviewer Expertise:

Infectious Diseases

I confirm that I have read this submission and believe that I have an appropriate level of expertise to confirm that it is of an acceptable scientific standard.

# Peer review: Reviewer response for version 2

**DOI:** 10.5256/f1000research.23885.r74702

**Published:** 2020-11-25

**Authors:** Prajjwal Pyakurel

**Affiliations:** 1 School of Public Health and Community Medicine, B.P. Koirala Institute of Health Sciences, Dharan, Nepal

1. First, during the Ebola outbreak, health facility visits for malaria, ARI/pneumonia and watery diarrhoea dropped significantly nation-wide, without returning to pre-Ebola levels post-outbreak. (Please explain the reason).
2. The outbreak was declared over in November 2015. Since then, there have been considerable investments into the health system by Government and development partners. (In terms of what?)
3. We thus conducted a similar country-wide study assessing morbidity and mortality for the same childhood illnesses using a longer post-Ebola period. (Can you specify what a longer period means?)
4. Is DHIS2 is the latest version? (Has it been upgraded?)
5. Could you discuss the transition period of recovery from Pre-Ebola to Ebola period and how the health system worked during that period so that the reader could correlate with your findings?

Is the work clearly and accurately presented and does it cite the current literature?

Yes

If applicable, is the statistical analysis and its interpretation appropriate?

Yes

Are all the source data underlying the results available to ensure full reproducibility?

Yes

Is the study design appropriate and is the work technically sound?

Yes

Are the conclusions drawn adequately supported by the results?

Yes

Are sufficient details of methods and analysis provided to allow replication by others?

Yes

Reviewer Expertise:

NA

I confirm that I have read this submission and believe that I have an appropriate level of expertise to confirm that it is of an acceptable scientific standard.

# Peer review: Reviewer response for version 2

**DOI:** 10.5256/f1000research.23885.r74706

**Published:** 2020-11-17

**Authors:** Alex Stewart

**Affiliations:** 1 College of Life and Environmental Science, University of Exeter, Exeter, UK

This is a revised version of a paper examining rates of consultations for childhood illnesses and deaths before, during and after the Ebola outbreak in Sierra Leone. The authors have responded fully to the reviewers' comments on the first submission.

 I have no further comment to make. The paper is clear, the results justify the conclusions, and there are considerable implications for health services both in Ebola and other outbreak and pandemic situations.

Is the work clearly and accurately presented and does it cite the current literature?

Yes

If applicable, is the statistical analysis and its interpretation appropriate?

Yes

Are all the source data underlying the results available to ensure full reproducibility?

Yes

Is the study design appropriate and is the work technically sound?

Yes

Are the conclusions drawn adequately supported by the results?

Yes

Are sufficient details of methods and analysis provided to allow replication by others?

Yes

Reviewer Expertise:

Public Health, Health Protection, Environmental public health, Iodine deficiency.

I confirm that I have read this submission and believe that I have an appropriate level of expertise to confirm that it is of an acceptable scientific standard.

# Peer review: Reviewer response for version 2

**DOI:** 10.5256/f1000research.23885.r58461

**Published:** 2020-01-30

**Authors:** Sara Hersey

**Affiliations:** 1 The World Bank, Washington, DC, USA

Is the work clearly and accurately presented and does it cite the current literature?

Partly

If applicable, is the statistical analysis and its interpretation appropriate?

Yes

Are all the source data underlying the results available to ensure full reproducibility?

Yes

Is the study design appropriate and is the work technically sound?

Partly

Are the conclusions drawn adequately supported by the results?

Partly

Are sufficient details of methods and analysis provided to allow replication by others?

Yes

Reviewer Expertise:

NA

I confirm that I have read this submission and believe that I have an appropriate level of expertise to confirm that it is of an acceptable scientific standard.

# Peer review: Reviewer response for version 1

**DOI:** 10.5256/f1000research.20306.r52585

**Published:** 2019-10-28

**Authors:** Justin Lessler

**Affiliations:** 1 Department of Epidemiology, Johns Hopkins Bloomberg School of Public Health, Baltimore, MD, USA

"Paediatric morbidity and mortality in Sierra Leone. Have things changed after the 2014/2015 Ebola outbreak?" provides a straight forward analysis of changes in reports of, and mortality from, four diseases before, during and after the 2014/2015 Ebola outbreak based on data from DHIS2 in Sierra Leone. The report provides simple, but important, statistics on morbidity and mortality for malaria, ARI/pneumonia, watery diarrhea, and measles. While the data and analysis is important, I have some significant concerns with the statistical analysis and interpretation of the results:

**Statistics:**

 While the use of chi-squared test to identify that significant differences in mortality exist is appropriate, it only is being used to compare pre- and post Ebola periods and does not help with evaluation of the size of the changes. I would suggest the authors instead select a reference period and report relative rates and confidence intervals, so the data can be better understood. Further, the authors do not perform statistical tests to support some of their statements, some of which are noted below. More tests for differences in reported rates between periods and some analysis of changes in the Ebola period would better support their conclusions.

**Interpretation:**

 The authors make several statements in the discussion and abstract that do not correspond to the analyses done. For instance, they make statements regarding trends in case reports, but perform no statistical test as to whether between period changes in reports are significant. Further, in some cases they frame speculation as a key finding of the paper, such as when they attribute changes to community confidence in the health system. Such speculations need to be clearly framed as such.

 Overall, this paper could be made far stronger and more valuable with the addition of just a few additional basic statistical analyses that were more aligned with the conclusions the authors which to highlight in the discussion. This analysis could likely be done while preserving the admirable conciseness and brevity of the paper. 

**Specific notes:    **

- Introduction, "to early to assess...": Could this not just be recovery, but also improvements, as significant investments followed the Ebola outbreak.
- Discussion, "First, the reductions in case fatality...": There is not really any evidence for this association in the paper, it should be, at the very least, more clear this is speculation. 
- Discussion, "Second, increased measles cases...": If this is going to be said it needs to be better supported by the statistical analysis in the paper. 
- Discussion, "In conclusion, consultations...": This was not explicitly tested in the analysis, so I am not sure it can be concluded.

Is the work clearly and accurately presented and does it cite the current literature?

Partly

If applicable, is the statistical analysis and its interpretation appropriate?

Partly

Are all the source data underlying the results available to ensure full reproducibility?

Yes

Is the study design appropriate and is the work technically sound?

Partly

Are the conclusions drawn adequately supported by the results?

No

Are sufficient details of methods and analysis provided to allow replication by others?

Yes

Reviewer Expertise:

Epidemiology,  statistics, infectious disease dynamics

I confirm that I have read this submission and believe that I have an appropriate level of expertise to confirm that it is of an acceptable scientific standard, however I have significant reservations, as outlined above.

# Response

**Published:** 2019-12-09

**Authors:** Tom Sesay

**Affiliations:** Ministry of Health and sanitation, Sierra Leone

**Author Response to Reviewer Report for Version 1 from Justin Lessler 28 Oct 2019**

Thank you for your thoughtful review of the manuscript and your suggestions. We have responded as below.

**1. Reviewer comment:** **"Paediatric morbidity and mortality in Sierra Leone. Have things changed after the 2014/2015 Ebola outbreak?" provides a straight forward analysis of changes in reports of, and mortality from, four diseases before, during and after the 2014/2015 Ebola outbreak based on data from DHIS2 in Sierra Leone. The report provides simple, but important, statistics on morbidity and mortality for malaria, ARI/pneumonia, watery diarrhea, and measles.**

**Response:**

Thank you for this comment.

**2. Reviewer comment:** **While the data and analysis is important, I have some significant concerns with the statistical analysis and interpretation of the results. Statistics: While the use of chi-squared test to identify that significant differences in mortality exist is appropriate, it only is being used to compare pre- and post Ebola periods and does not help with evaluation of the size of the changes. I would suggest the authors instead select a reference period and report relative rates and confidence intervals, so the data can be better understood. Further, the authors do not perform statistical tests to support some of their statements, some of which are noted below. More tests for differences in reported rates between periods and some analysis of changes in the Ebola period would better support their conclusions.**

**Response:**

Thank you for this point. We have performed t-tests for the differences in the mean monthly cases of the intra-Ebola and post-Ebola periods relative to the pre-Ebola period at a level of significance of 5%. We have also updated the figures by disease condition, which better displays the differences between the periods.

**3. Reviewer comment:** **Interpretation: The authors make several statements in the discussion and abstract that do not correspond to the analyses done. For instance, they make statements regarding trends in case reports, but perform no statistical test as to whether between period changes in reports are significant. Further, in some cases they frame speculation as a key finding of the paper, such as when they attribute changes to community confidence in the health system. Such speculations need to be clearly framed as such.**

**Response:**

We have taken note of this comment and, accordingly, removed comments that could be considered as speculations.

**4. Reviewer comment:** **Overall, this paper could be made far stronger and more valuable with the addition of just a few additional basic statistical analyses that were more aligned with the conclusions the authors which to highlight in the discussion. This analysis could likely be done while preserving the admirable conciseness and brevity of the paper.**

**Response:**

We have included the basic statistical tests to test the statistical significance of differences between periods, as mentioned above.

    

**5. Reviewer comment:** **Introduction, "to early to assess...": Could this not just be recovery, but also improvements, as significant investments followed the Ebola outbreak.**

**Response:**

This comment is noted, and we have added in a phrase on possible improvements.

**6. Reviewer comment:** **Discussion, "First, the reductions in case fatality...” There is not really any evidence for this association in the paper, it should be, at the very least, more clear this is speculation.**

**Response:**

The reductions in case fatality were indicated by a Chi-square test of the difference between the pre-Ebola and the post-Ebola period which showed a significant reduction in case fatality in the post- Ebola period.

**7. Reviewer comment:** **Discussion, "Second, increased measles cases...": If this is going to be said it needs to be better supported by the statistical analysis in the paper.**

**Response:**

We have included a t-test of the average monthly measles cases, which showed significant increase in the number of measles cases between the pre-Ebola and post-Ebola periods.

**8. Reviewer comment:** **Discussion, "In conclusion, consultations...": This was not explicitly tested in the analysis, so I am not sure it can be concluded.**

**Response:**

We have ensured that the relevant statistical tests have been completed, with corresponding results to support the conclusions.

# Peer review: Reviewer response for version 1

**DOI:** 10.5256/f1000research.20306.r52581

**Published:** 2019-10-08

**Authors:** Sara Hersey

**Affiliations:** 1 The World Bank, Washington, DC, USA

The authors of this paper have looked at trends in the pediatric morbidity and mortality prior to, during and after the 2014-15 Ebola outbreak in West Africa. The impact of large scale outbreaks on health systems remains a critical area of research, and the authors should be congratulated in contributing to this area of work. The comments below are intended to better contextualize the analysis and conclusions proposed by the authors.

- The authors divide the paper into three distinct time periods, with ‘post-Ebola’ defined as beginning in June 2016 and extending through April 2017. While the end of the Ebola outbreak in Sierra Leone was declared in November 2015, there continued to be additional Ebola clusters in the country and the region into the ‘post-Ebola’ study period. The policy of mandatory Ebola testing for all deaths also continued until June 2016.
    
- During the ‘post-Ebola’ time period in the paper, there was an enormous influx of funding to improve health systems at both the facility and community levels that was organized under the Presidential Recovery Plan. This funding supported the national and sub-national health systems but was also supplemented by NGO and other non-governmental health services. By the end of 2017, there were substantial reductions in the funds available for health systems and service delivery.
- The continued specter of Ebola transmission and the rapid scale-up in health resources likely had both negative and positive impacts on health systems utilization that are difficult to quantify. The authors have documented significantly lower case fatality rates for three diseases than the pre-Ebola time period which would support the conclusion that there was regained community trust in the health services. However, those services were not the same ones available prior to the Ebola outbreak and the services are difficult to maintain with reduced external resources.
- In order to compare the pre and post-Ebola periods, I would recommend that the analysis be extended to the immediate post-Ebola period included in this paper as well as a longer-term post-Ebola period through 2018 which would better capture the more sustainable health system in Sierra Leone rather than the one served by a short-term, high-volume influx of external funding.
- Aside from the clear spikes in measles consultations, Figure 1 is difficult to interpret when displayed by time period. The differences may be more impactful if compared by disease instead.
- The analysis supports the conclusion that measles surged during and post-Ebola and this is backed up by other research and program documentation. However, it does not necessarily support the recommendation to extend the cut off age for vaccination. The recommendation to conduct laboratory confirmation for measles is also not necessarily supported by the data presented. Is this because a number of the measles outbreaks were determined to be rubella which is currently not on the vaccine schedule? If yes, this should be documented by the data. What would be the impact on measles morbidity if there was increased diagnostic versus syndromic management of measles?

Is the work clearly and accurately presented and does it cite the current literature?

Partly

If applicable, is the statistical analysis and its interpretation appropriate?

Yes

Are all the source data underlying the results available to ensure full reproducibility?

Yes

Is the study design appropriate and is the work technically sound?

Partly

Are the conclusions drawn adequately supported by the results?

Partly

Are sufficient details of methods and analysis provided to allow replication by others?

Yes

Reviewer Expertise:

Infectious disease epidemiology

I confirm that I have read this submission and believe that I have an appropriate level of expertise to confirm that it is of an acceptable scientific standard, however I have significant reservations, as outlined above.

# Response

**Published:** 2019-12-09

**Authors:** Tom Sesay

**Affiliations:** Ministry of Health and sanitation, Sierra Leone

**Author Response to Reviewer Report for Version 1 from Sara Hersey 08 Oct 2019**

Thank you for your thoughtful review of the manuscript and your insightful suggestions. We have responded as below.

**1. Reviewer comment:** **The authors of this paper have looked at trends in the pediatric morbidity and mortality prior to, during and after the 2014-15 Ebola outbreak in West Africa. The impact of large scale outbreaks on health systems remains a critical area of research, and the authors should be congratulated in contributing to this area of work. The comments below are intended to better contextualize the analysis and conclusions proposed by the authors.**

**Response:**

Thank you for recognising the importance of this study and its contribution to knowledge about the impact of the 2014/15 Ebola outbreak.

**2. Reviewer comment:** **The authors divide the paper into three distinct time periods, with ‘post-Ebola’ defined as beginning in June 2016 and extending through April 2017. While the end of the Ebola outbreak in Sierra Leone was declared in November 2015, there continued to be additional Ebola clusters in the country and the region into the ‘post-Ebola’ study period. The policy of mandatory Ebola testing for all deaths also continued until June 2016.**

****

**During the ‘post-Ebola’ time period in the paper, there was an enormous influx of funding to improve health systems at both the facility and community levels that was organized under the Presidential Recovery Plan. This funding supported the national and sub-national health systems but was also supplemented by NGO and other non-governmental health services. By the end of 2017, there were substantial reductions in the funds available for health systems and service delivery.**

****

**The continued specter of Ebola transmission and the rapid scale-up in health resources likely had both negative and positive impacts on health systems utilization that are difficult to quantify. The authors have documented significantly lower case fatality rates for three diseases than the pre-Ebola time period which would support the conclusion that there was regained community trust in the health services. However, those services were not the same ones available prior to the Ebola outbreak and the services are difficult to maintain with reduced external resources.**

****

**In order to compare the pre and post-Ebola periods, I would recommend that the analysis be extended to the immediate post-Ebola period included in this paper as well as a longer-term post-Ebola period through 2018 which would better capture the more sustainable health system in Sierra Leone rather than the one served by a short-term, high-volume influx of external funding.**

**Response:**

Thank you for this recommendation which we do understand and accept. We do acknowledge the need for further research into the sustainability of the early post-Ebola gains in a much longer post-Ebola period. However, the objective of this study was to assess whether the investments in the health system had an impact on service delivery in the post-Ebola period, as defined in the manuscript. In addition, the ethical approval obtained for this study was only for the periods mentioned in the paper. This, however, is an area we will be interested to investigate in the near future. We have added a recommendation to this effect in the discussion section.

**3. Reviewer comment:** **Aside from the clear spikes in measles consultations, Figure 1 is difficult to interpret when displayed by time period. The differences may be more impactful if compared by disease instead.**

**Response:**

We agree with your recommendation, and accordingly have completed the analysis separately by the disease conditions. We have amended the Figure 1 based on this.

**4. Reviewer comment:** **The analysis supports the conclusion that measles surged during and post-Ebola and this is backed up by other research and program documentation. However, it does not necessarily support the recommendation to extend the cut off age for vaccination. The recommendation to conduct laboratory confirmation for measles is also not necessarily supported by the data presented. Is this because a number of the measles outbreaks were determined to be rubella which is currently not on the vaccine schedule? If yes, this should be documented by the data. What would be the impact on measles morbidity if there was increased diagnostic versus syndromic management of measles?**

**Response:**

We do agree with this recommendation. We have removed the recommendation to extend the cut off age for vaccination and added the recommendation to increase the coverage of the second measles dose at 15 months.
